# Suture Technique for Fascial Closure in Midline Laparotomy in Patients at High Risk of Post-surgical Evisceration

**DOI:** 10.7759/cureus.113530

**Published:** 2026-07-28

**Authors:** Luis Eduardo Morillo Lemus, Ruben Lopez Olivares

**Affiliations:** 1 Surgery, Clinica Abasolo, Moroleon, MEX; 2 Surgery, Hospital Regional De Pemex Poza Rica, Poza Rica, MEX

**Keywords:** aponeurotic closure, midline laparotomy, postsurgical evisceration, second-level care hospital, wall closure

## Abstract

The objective of this study is to describe the modified Maingot keel closure technique as a reinforced fascial closure strategy in patients at high risk of postoperative evisceration and to report the short-term clinical outcomes associated with its application.

We present a case series of four patients undergoing midline laparotomy who were considered at high risk for postoperative fascial dehiscence according to a Van Ramshorst score greater than 6. The modified Maingot keel closure technique was performed as primary or secondary fascial reinforcement according to the clinical scenario.

Four patients (three women and one man) older than 50 years were included. The Van Ramshorst scores ranged from 7 to 9. The mean operative time was approximately three hours. No intraoperative complications, postoperative evisceration, surgical reintervention, or wound-related complications were observed during follow-up. The mean hospital stay was 4.5 days.

The modified Maingot keel closure technique was technically feasible and was associated with favorable short-term outcomes in this selected group of high-risk patients. However, due to the limited sample size and absence of a comparison group, these findings should be interpreted cautiously. Prospective comparative studies with larger populations and longer follow-up are required to determine its definitive role in abdominal wall closure strategies.

## Introduction

Postoperative fascial dehiscence following midline laparotomy is an uncommon but potentially life-threatening complication associated with increased morbidity, mortality, prolonged hospitalization, and healthcare costs. This complication occurs due to failure of fascial healing and may progress to postoperative evisceration, which requires urgent clinical management because of the risk of bowel injury, infection, and reoperation.

The reported incidence of postoperative fascial dehiscence varies according to patient characteristics, surgical indication, and perioperative risk factors. Previous studies have reported higher rates among patients undergoing emergency laparotomy and those with multiple risk factors, including advanced age, malnutrition, obesity, infection, increased intra-abdominal pressure, and systemic comorbidities.

Several strategies have been evaluated to reduce the incidence of abdominal wall failure. These include modifications in fascial closure techniques, optimization of suture length-to-wound length ratio, small-bites closure techniques, and prophylactic mesh reinforcement in selected high-risk patients. Although prophylactic mesh has demonstrated preventive benefits in certain populations, concerns remain regarding patient selection, contamination risk, foreign material implantation, and potential complications. Therefore, an optimal universally accepted strategy for all high-risk patients has not yet been established.

Risk prediction models, such as the Van Ramshorst score, have been developed to identify patients with increased probability of postoperative evisceration. Patients with scores greater than 6 represent a subgroup with substantially increased risk and may benefit from individualized preventive approaches.

The modified Maingot keel closure technique is a reinforced fascial closure method designed to improve abdominal wall strength by combining interrupted absorbable keel sutures with additional continuous non-absorbable reinforcement. Unlike conventional continuous fascial closure, this technique aims to distribute tension across multiple fixation points while providing additional mechanical support to the abdominal wall. This configuration may reduce focal stress concentration at the fascial edges and provide additional resistance against mechanical failure during the early postoperative healing period.

The technique was initially described for incisional hernia repair and later adapted as a preventive closure strategy in patients considered at high risk for postoperative fascial failure. However, available evidence remains limited, and additional clinical descriptions are required to evaluate its feasibility and short-term outcomes in selected high-risk patients.

Therefore, the objective of this study is to describe the application of the modified Maingot keel closure technique in patients at high risk of postoperative evisceration and report the short-term clinical outcomes associated with this approach.

## Case presentation

We present four patients undergoing midline laparotomy who were considered at high risk for postoperative fascial dehiscence according to a Van Ramshorst score greater than 6.

Patients were selected based on the presence of multiple risk factors associated with abdominal wall failure. All patients were older than 50 years and underwent open abdominal surgery requiring midline fascial closure.

For each patient, demographic characteristics, underlying surgical diagnosis, surgical indication, emergency or elective status, relevant risk factors included in the Van Ramshorst score, operative time, postoperative complications, length of hospital stay, and follow-up outcomes were recorded.

The demographic characteristics, surgical indications, risk factors, operative details, and postoperative outcomes are summarized in Table 1.

**Table 1:** Clinical characteristics and postoperative outcomes of patients undergoing modified Maingot keel closure technique

Patient	Age group	Sex	Surgical indication/clinical scenario	Type of modified Maingot keel closure	Van Ramshorst score	Operative time	Hospital stay	Postoperative complications	Follow-up outcome
Patient 1	50-60 years	Female	Midline laparotomy in a patient at high risk for fascial dehiscence	Primary closure	7	3 hours	4 days	None reported	No evisceration or fascial dehiscence
Patient 2	60-70 years	Female	Midline laparotomy in a patient at high risk for fascial dehiscence	Primary closure	8	3 hours	4 days	None reported	No evisceration or fascial dehiscence
Patient 3	60-70 years	Female	Midline laparotomy in a patient at high risk for fascial dehiscence	Primary closure	7	2 hours	2 days	None reported	No evisceration or fascial dehiscence
Patient 4	70-80 years	Male	Postoperative evisceration after previous fascial closure failure	Secondary reinforcement closure	9	4 hours	8 days	None reported after reinforcement	No recurrent evisceration

In three patients, the modified Maingot keel closure technique was performed as the initial fascial closure method because of the high risk of postoperative evisceration. In one patient, the technique was used as a secondary reinforcement procedure after postoperative evisceration caused by previous fascial closure failure.

The technique consisted of reinforced fascial approximation using keel sutures designed to distribute tension along the midline laparotomy closure. During follow-up, no patients developed recurrent fascial dehiscence, postoperative evisceration, or required surgical reintervention.

The modified Maingot keel closure technique was performed either as primary reinforcement during the initial laparotomy closure or as a secondary intervention after failure of previous fascial closure in a patient presenting with postoperative evisceration.

**Figure 1:**
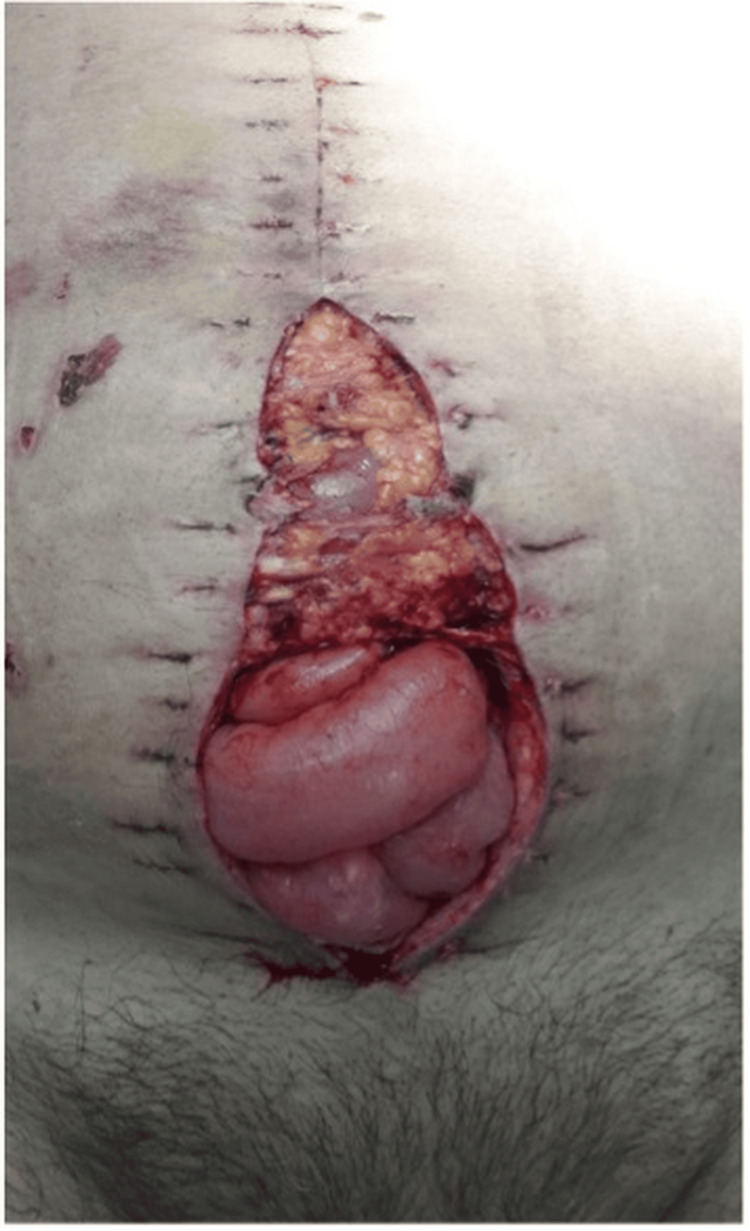
Patient presenting with postoperative evisceration after failure of primary fascial closure. The image demonstrates separation of the abdominal fascia with exposure and protrusion of intra-abdominal contents through the laparotomy incision. This case represents the clinical scenario that motivated the use of reinforced fascial closure strategies in high-risk patients.

**Figure 2:**
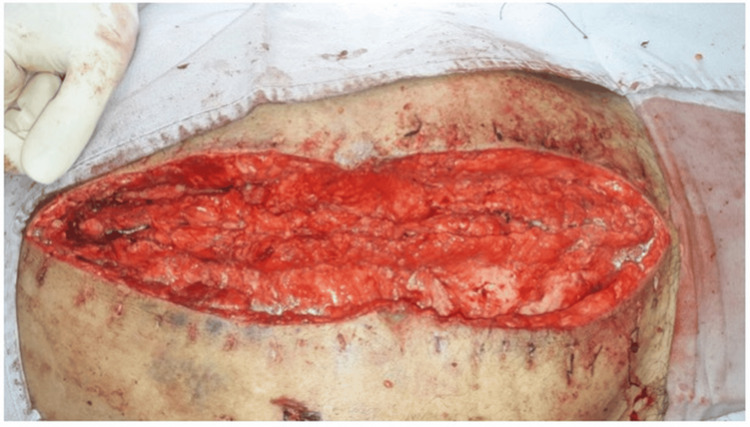
Modified Maingot keel fascial closure technique The image demonstrates the placement of interrupted absorbable keel sutures providing reinforcement of the fascial edges. The technique creates multiple fixation points designed to distribute tension along the midline laparotomy closure.

**Figure 3:**
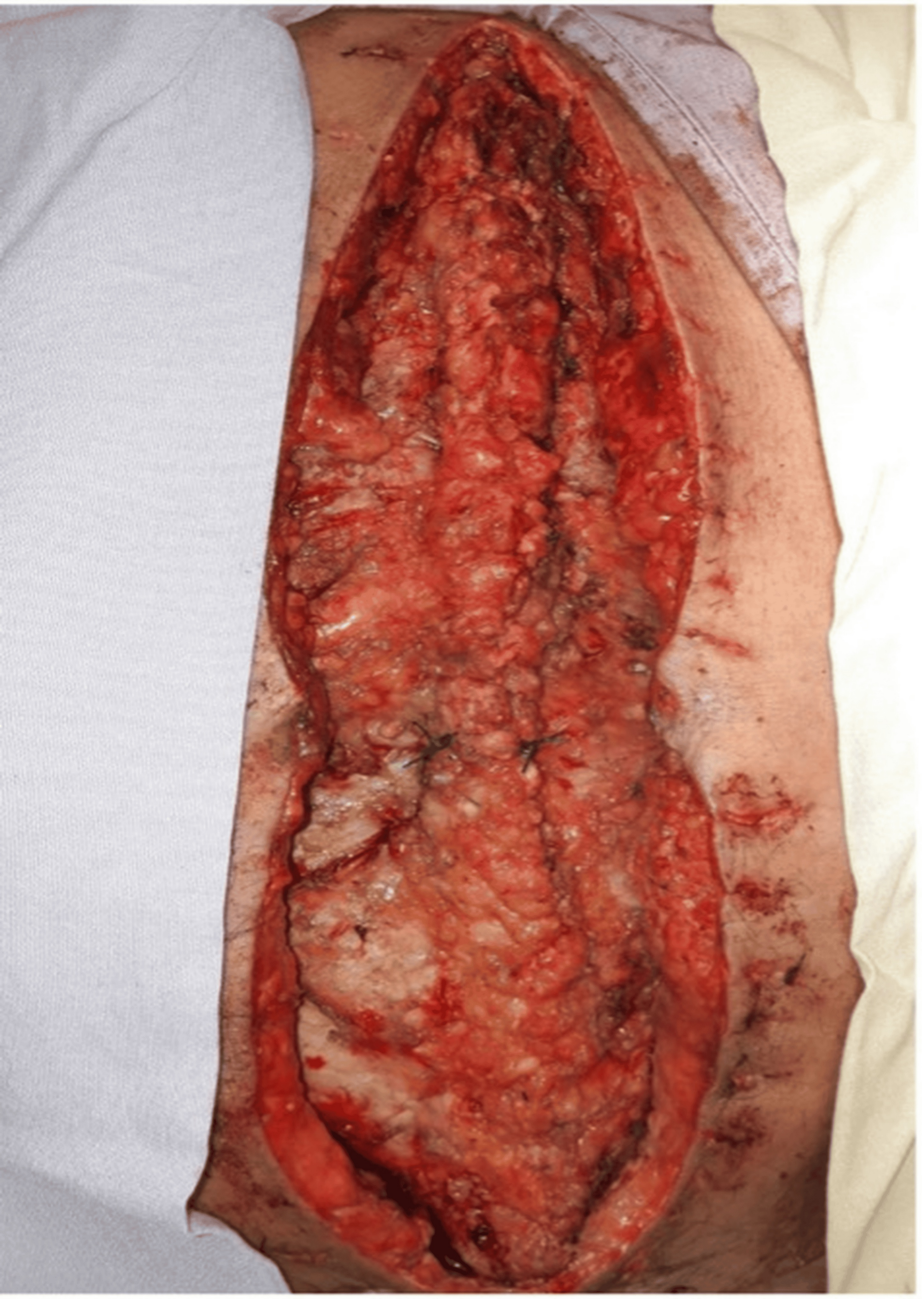
Reinforcement of closure using continuous non-absorbable running suture

No patient required reoperation due to recurrent evisceration during follow-up. Clinical outcomes were assessed based on wound integrity, postoperative complications, and evidence of fascial failure (Figures 1-3).

**Figure 4:**
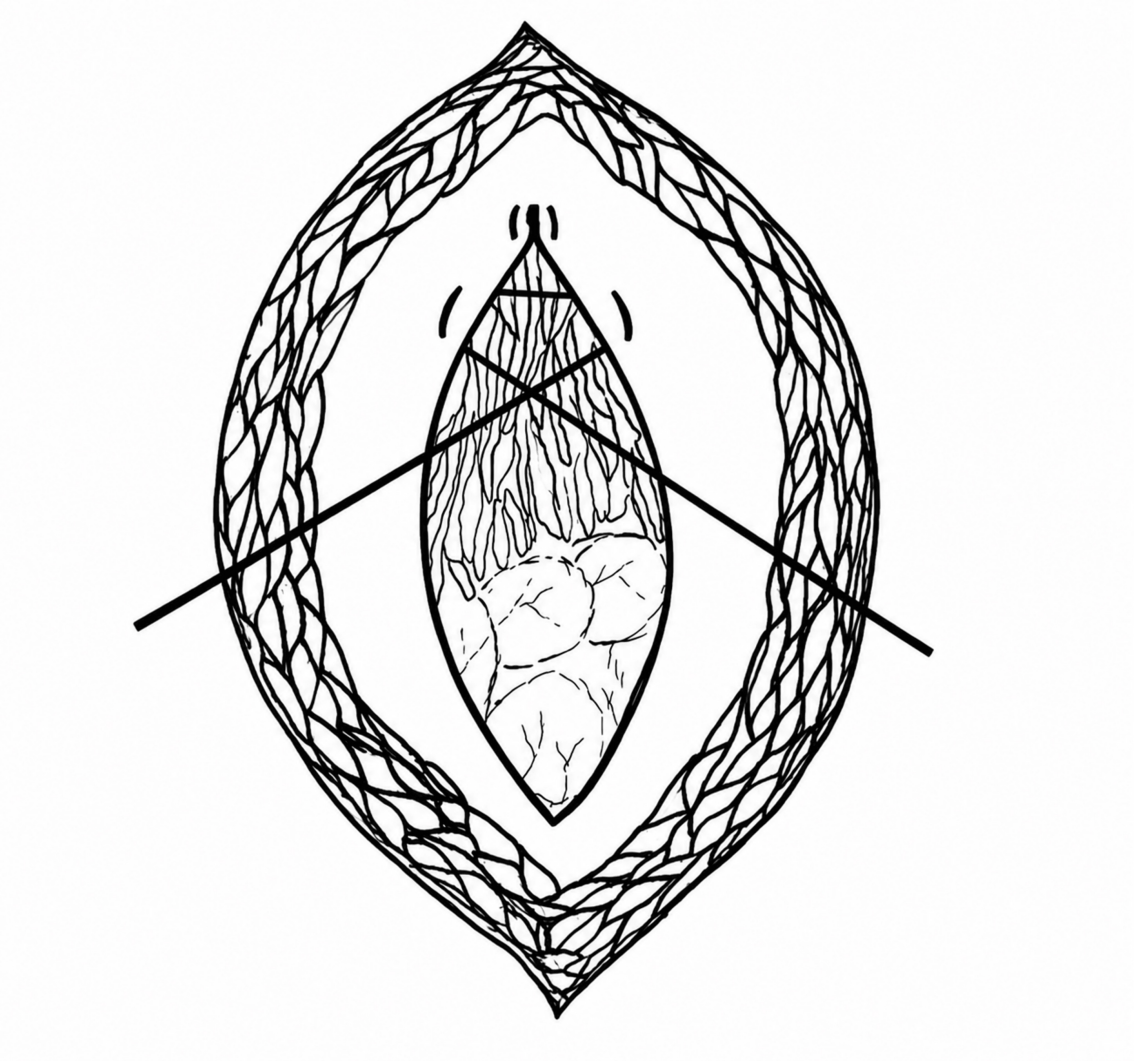
The diagram illustrates the passage of interrupted keel sutures through the fascial edges, showing the inward folding configuration that provides reinforcement and tension distribution along the closure line. The image was created using the GoodNotes 6 application (Time Base Technology Limited, Hong Kong) on an iPad.

**Figure 5:**
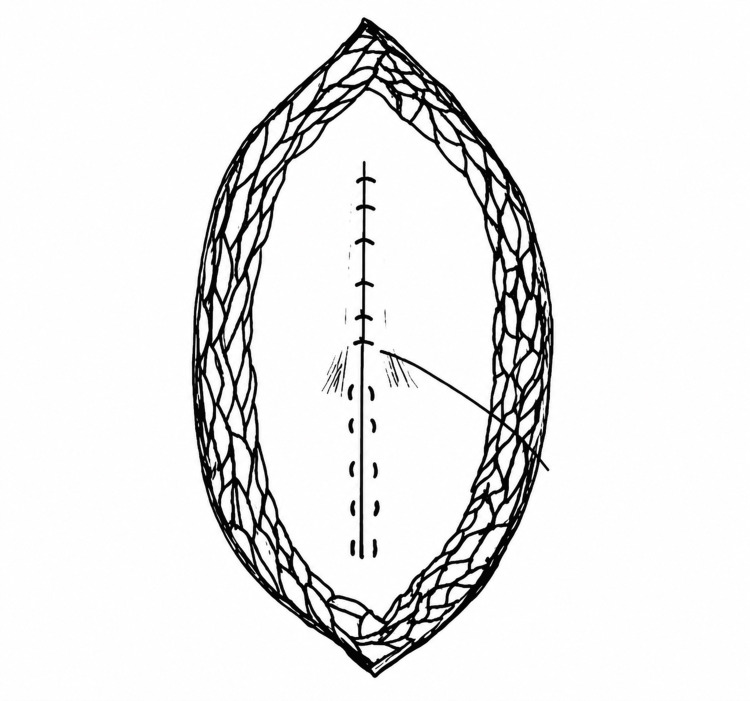
The final configuration demonstrates reinforcement of the keel closure with a continuous non-absorbable running suture placed over the interrupted fascial sutures. The image was created using the GoodNotes 6 application (Time Base Technology Limited, Hong Kong) on an iPad.

In all four patients in whom primary fascial closure was considered technically difficult, a standardized closure protocol was applied as follows: Macroscopic control of the surgical field was ensured, with removal of any residual septic or contaminated tissue depending on the primary procedure performed. Radical debridement of wound edges was carried out, excising all devitalized and contaminated tissue to achieve clearly viable and healthy margins suitable for closure. Anesthetic relaxation of the abdominal wall was requested during fascial closure to facilitate approximation and reduce tension. Interrupted fascial sutures were placed using the Maingot keel technique with non-absorbable No. 1 suture material (Figures 4, 5).

All sutures were subsequently crossed and progressively tightened to achieve maximal approximation of the aponeurotic edges. Finally, reinforcement of the closure was performed using a continuous running suture over the initial keel-layer repair. This combined technique was used as a reinforced abdominal wall closure strategy in all selected high-risk patients. 

## Discussion

Postoperative fascial dehiscence after midline laparotomy remains one of the most concerning complications in abdominal surgery because of its association with increased morbidity, prolonged hospitalization, need for reoperation, and the possibility of postoperative evisceration. Although improvements in surgical technique and perioperative management have reduced its incidence, fascial failure continues to represent an important clinical challenge, particularly among patients with multiple risk factors affecting tissue healing and abdominal wall integrity [1].

The development of fascial dehiscence is considered a multifactorial process involving patient-related conditions, surgical factors, and mechanical properties of the abdominal wall. Advanced age, emergency surgery, anemia, pulmonary disease, wound infection, and impaired nutritional status have been identified as important contributors to poor fascial healing [1,2]. In this context, risk stratification tools such as the Van Ramshorst score may help identify patients at increased risk who may benefit from individualized preventive strategies [1]. 

In the present case series, we describe the application of the modified Maingot keel closure technique in four patients classified as high risk for postoperative evisceration according to a Van Ramshorst score greater than 6. The main observation was that the technique was technically feasible and was associated with favorable short-term outcomes, with no cases of postoperative evisceration, fascial disruption, or surgical reintervention during follow-up. However, these findings should be interpreted as preliminary observations regarding feasibility and safety and should not be considered evidence of superiority compared with other closure techniques.

Several approaches have been evaluated to reduce the risk of abdominal wall failure after laparotomy. Conventional fascial closure techniques have been modified over time, with emphasis on improving tension distribution and reducing excessive stress on the tissue. The use of appropriate suture technique and adequate suture length has been associated with improved abdominal wall outcomes and reduced risk of incisional complications [3]. 

The small-bites technique has represented an important modification in abdominal wall closure. Randomized clinical studies have demonstrated that small-bites closure may decrease the incidence of incisional hernia compared with traditional large-bites closure by distributing mechanical forces more evenly along the incision [4]. Similarly, studies evaluating the suture length-to-wound length ratio have demonstrated that closure technique characteristics influence the mechanical resistance of the abdominal wall and may affect postoperative outcomes [5]. 

Another preventive strategy that has been explored in high-risk patients is prophylactic mesh reinforcement. Randomized studies have demonstrated that mesh reinforcement can reduce the incidence of incisional hernia after laparotomy in selected populations. However, the use of prosthetic material remains controversial because of concerns regarding infection, foreign-body reaction, cost, and applicability in contaminated surgical fields [6]. Current recommendations emphasize individualized decision-making based on patient risk factors, surgical contamination, and expected benefits [7].

Patient optimization also plays an essential role in preventing wound complications. Nutritional impairment has been associated with delayed wound healing due to alterations in collagen synthesis, immune response, and tissue regeneration. Therefore, nutritional assessment and optimization should be considered important components of perioperative management, particularly in patients undergoing major abdominal surgery [8].

In addition to nutritional factors, prevention of surgical site infection remains fundamental in reducing wound complications. Infection negatively affects tissue repair, increases collagen degradation, and may contribute to fascial failure. International guidelines emphasize the importance of evidence-based preventive measures, including appropriate antimicrobial prophylaxis, surgical technique optimization, and perioperative management strategies [9]. 

The modified Maingot keel closure technique represents a reinforced fascial closure strategy designed to increase mechanical support through multiple fixation points. Unlike conventional closure methods, this approach provides additional reinforcement through keel sutures that distribute tension along the fascial edges. Although keel-type techniques have been described mainly in abdominal wall reconstruction and incisional hernia repair, their application as a preventive strategy in patients at high risk of postoperative evisceration remains insufficiently studied [10]. 

In our series, the modified Maingot keel closure technique was successfully performed in all patients without intraoperative complications or early postoperative fascial failure. These findings suggest that the technique may represent a feasible alternative reinforcement strategy in selected patients where additional fascial support is considered necessary. Nevertheless, this study does not establish whether the technique reduces the incidence of postoperative evisceration or provides superior outcomes compared with conventional closure methods or mesh reinforcement.

The interpretation of these findings must consider several limitations. The small sample size, descriptive case-series design, absence of a control group, and limited follow-up prevent definitive conclusions regarding effectiveness. Additionally, the selected nature of the patient population and the performance of the procedure by a specialized surgical team may limit generalizability. Future prospective comparative studies with larger populations and longer follow-up are required to determine the role of the modified Maingot keel closure technique in abdominal wall closure strategies.

Limitations

This study has several limitations. First, the small sample size and descriptive case-series design limit the ability to establish causal relationships or determine the effectiveness of the modified Maingot keel closure technique. Second, the absence of a control group prevents direct comparison with conventional closure methods or other reinforcement strategies. Third, follow-up duration was limited, preventing evaluation of late complications such as incisional hernia formation. Finally, all procedures were performed in a selected high-risk population by a specialized surgical team, which may limit the generalizability of these findings.

Because this was a retrospective descriptive case series, only variables available in the original clinical records were analyzed. Detailed individual comorbidity profiles and long-term follow-up variables were not available for all patients.

## Conclusions

Postoperative evisceration remains a severe complication after midline laparotomy, particularly among high-risk patients. The modified Maingot keel closure technique showed favorable short-term outcomes in this case series, without postoperative evisceration, increased morbidity, or prolonged operative time.

Nevertheless, these findings should be interpreted cautiously due to the limited sample size. Further prospective comparative studies with longer follow-up are necessary to determine its effectiveness compared with conventional closure methods and mesh reinforcement strategies.

## References

[1] García-Montero A, Viedma-Contreras S, Martínez-Blanco N, Gombau-Baldrich Y, Guinot-Bachero J (2018). Multidisciplinary approach to infected abdominal dehiscence: cost-consequence evaluation of dressings and measures used. Gerokomos.

[2] Gili-Ortiz E, González-Guerrero R, Béjar-Prado L, Ramírez-Ramírez G, López-Méndez J (2015). Dehiscencia de la laparotomía y su impacto en la mortalidad, la estancia y los costes hospitalarios. Cir Esp.

[3] van Ramshorst GH, Nieuwenhuizen J, Hop WC, Arends P, Boom J, Jeekel J, Lange JF (2010). Abdominal wound dehiscence in adults: development and validation of a risk model. World J Surg.

[4] Xing L, Culbertson EJ, Wen Y, Franz MG (2013). Early laparotomy wound failure as the mechanism for incisional hernia formation. J Surg Res.

[5] Sánchez-Fernández P, Mier y Díaz J, Castillo-González A, Blanco-Benavides R, Zárate-Castillo J (2000). Factores de riesgo para dehiscencia de herida quirúrgica. Cir Ciruj.

[6] Deerenberg E, Harlaar J, Steyerberg E (2015). Small bites versus large bites for closure of abdominal midline incisions (STITCH): a double-blind, multicentre, randomised controlled trial. Lancet.

[7] Alrashidi A, Hageen AW, Rifai M (2025). Small bites versus large bites for closure of midline abdominal incisions: a systematic review and meta-analysis with trial sequential analysis of randomized controlled trials. World J Surg.

[8] Muysoms FE, Antoniou SA, Bury K (2015). European Hernia Society guidelines on closure of abdominal wall incisions. Hernia.

[9] Dodds SR, Finch D, Chant AD (1997). Early effect of carotid endarterectomy on arterial blood pressure measured with an ambulatory monitor. Br J Surg.

[10] Cardoso MJ, Sciubba DM (2009). Is use of bone-morphogenetic proteins for spine fusion surgery cost-effective?. Arch Surg.

